# Complex motor imagery in elite female ice hockey players: a cortical arena of imagination revealed by magnetoencephalography

**DOI:** 10.3389/fnhum.2026.1754371

**Published:** 2026-02-27

**Authors:** Audrey Alice Potts, Luis Garcia Dominguez, David Gold, Mary Pat McAndrews, Richard Wennberg

**Affiliations:** 1 Mitchell Goldhar Magnetoencephalography (MEG) Unit, Krembil Brain Institute, University Health Network, Toronto, ON, Canada; 2 Department of Psychiatry, University of Toronto, Toronto, ON, Canada; 3 Division of Clinical and Computational Neuroscience, Krembil Brain Institute, University Health Network, Toronto, ON, Canada; 4 Department of Psychology, University of Toronto, Toronto, ON, Canada; 5 Division of Neurology, Department of Medicine, University of Toronto, Toronto, ON, Canada

**Keywords:** cognition, dreaming, MEG, neuroscience, sport, visualization

## Abstract

Motor imagery (MI), or “visualization,” as practiced by elite athletes to improve performance, provides a model of how covert thought—imagination—can affect subsequent behavior. In this exploratory magnetoencephalography (MEG) study, we aimed to identify the brain regions involved in complex MI in a small sample of elite female ice hockey players experienced in visualization. Using an experimental block design, the athletes visualized a specific PETTLEP (physical, environment, task, timing, learning, emotion, perspective)-guided scripted ice hockey play while being monitored with MEG. A frequency-domain beamformer was then calculated to contrast the MEG data from the imagery condition with two different control (resting state or mental counting) conditions. Significance was assessed using a cluster-based permutation test. The beamforming results identified a principal hub of neural activity during the imagery condition in a posterior left hemisphere cortical region surrounding the intraparietal sulcus. The same brain region was reliably activated in all eight participants and may hypothetically demarcate the neural substrate of this type of conscious thought.

## Introduction

Sometimes referred to in sports as “visualization,” motor imagery (MI) involves the imagining and vivid mental rehearsal of movements without overt motor output (Moran et al., 2012). It is commonly classified according to predominant type as kinesthetic (“feeling”) or visual (“seeing”), with visual taking either the first- or third-person perspective. In practice, multiple modalities may be used concurrently. Psychological evidence for the use and effectiveness of visualization in sports is well established (Weinberg, 2008). Early sport psychology research focused on methods for optimizing MI practices to maximize performance, leading to the development of the well-known kinesthetic method referred to by the acronym PETTLEP (Holmes and Collins, 2001). Conceptualized as a neuroscientific approach to MI in sport, the PETTLEP model proposed that seven key features—physical, environment, task, timing, learning, emotion, and perspective—be incorporated into an athlete’s imagery practices to be most effective. Grounded in the theory of functional equivalence, which presumes that overt and covert movement share similar patterns of activation in the brain (Lang, 1979; Jeannerod, 1995; Kraeutner et al., 2014), parallel to visual imagery and perception (Farah, 1989; Pearson et al., 2015), PETTLEP has been widely adopted as a tool in sport psychology, although questions persist regarding underlying neurophysiological mechanisms and the concept of neuroanatomical functional equivalence (Wakefield et al., 2013).

For “open” sports, defined by unpredictable environments and a need for athletes to react rapidly to complex behaviors, visualization of potential scenarios requires a multimodal approach to MI that integrates manifold aspects of human perception, experience, and action. Ice hockey is a fast, open team sport in which players make rapid decisions in response to an infinite number of different situations, decisions based on cognitive—conscious or subconscious—associations between multiple types of sensory stimuli and motor schema. One might intuitively expect that MI would be most useful in “closed” sports (e.g., diving, figure skating, and golf), and experimental studies of MI have commonly investigated simple movements (e.g., sequential finger tapping and object grasping) in a laboratory setting or, in sport-specific contexts, have focused on closed sport actions (e.g., golf swing) (Decety et al., 1994; Ross et al., 2003; Guillot et al., 2008; Kraeutner et al., 2014).

We were particularly interested in the more complex form of MI used by open sport athletes, where PETTLEP-based “scripts” of multifaceted scenarios are used as the foundation for an athlete’s imagery practice. At a fundamental level, complex MI provides a model of how covert thought—imagination—can affect subsequent behavior, a mind–brain phenomenon that presents an opportunity to explore the relationship between consciousness and action (Moran et al., 2012). The specific goal of this exploratory study was to determine if the brain region(s) most involved in sustaining this form of dynamic imagination could be revealed by magnetoencephalography (MEG).

## Materials and methods

The script used in this study of eight elite female ice hockey players incorporated all PETTLEP features with as much detail as possible (see Appendix: Visualization Script). Using an experimental block design, the athletes visualized a specific 2-on-1 ice hockey play (a goaltender used a different script) while their brain activity was monitored using MEG. In brief, 3-min baseline recording (resting state, no task) was followed by 10 trials of 30 s of imagery, alternating with 30 s of a mental counting task. MEG beamforming was then used to contrast the data from the imagery condition with the two different control (resting state or mental counting) conditions.

### Participants

A convenience sample of eight right-handed female elite ice hockey players (age range 20–26 years, active or less than 2 years retired at the time of study) was recruited from the Canadian Women’s Hockey League, the Canadian Women’s National Ice Hockey Team, the National Collegiate Athletic Association, or U Sports to participate in the study. All of the athletes were experienced practitioners of complex sport-specific MI. Participants rehearsed their imagery script the night before and/or the morning of the MEG recording, and all of them reported that they felt comfortable with the script and confident in their ability to imagine it. Participants provided written informed consent, and the study was approved by the local institutional Research Ethics Board (19-5,755).

### Study design

There were three parts to the study. (1) The Movement Imagery Questionnaire—Revised second version (MIQ-RS): completed by all participants to assess baseline visual and kinesthetic (seeing and feeling) imagery ability (see Supplementary material). (2) A 13-min MEG recording (after ~20 min of set up and calibration): participants underwent MEG recording inside a magnetically shielded room, beginning with 3 min of baseline resting state acquisition, followed by 10 30-s trials during which they performed the MI/visualization procedure, alternating with 10 30-s trials of a control task wherein participants performed a mental counting exercise (counting up by 3s “in their head”). Auditory “GO” cues triggered the 30-s visualization trials, and auditory “STOP” cues triggered the 30-s mental counting trials. (3) A structural magnetic resonance imaging (MRI) scan of the brain.

For this exploratory study, it was not clear what the optimal control condition would be to characterize the core components of the MI task. Areas of intrinsic network activity that may be detected at rest include bilateral sensorimotor and default mode regions, both of which might be expected to overlap with regions engaged by the MI task, given the frequent occurrence of motor imagination and episodic simulation (“day-dreaming”) during the resting state. Conversely, an active control task, such as mental counting, necessitates sustained engagement and attention but would be less likely to engage the visuomotor, executive, or emotional components of the MI task. Thus, we decided to conduct the same analyses with each of these controls.

### Motor imagery

As aforementioned, the specific ice hockey visualization procedure that participants imagined upon hearing the “GO” cues during their MEG recordings was created using the PETTLEP guide to best mimic the real-life experience (see Appendix).

### MEG recordings

Recordings were acquired using an Elekta Neuromag TRIUX 306-channel MEG system (Helsinki, Finland). Sampling frequency was 1,000 Hz with an online filter bandwidth of 0.1–330 Hz. Offline data processing commenced with artifact suppression using the default parameters (10-s time window, subspace correlation: 0.980) of the spatiotemporal signal space separation algorithm and head movement compensation, as implemented within the Elekta MaxFilter suite. Head position inside the MEG sensor array was determined from five coils attached to the scalp; a Polhemus 3D-Fastrak system (Colchester, United States) was used to digitize head shape and location of the head position indicator coils.

### MEG analyses

#### Forward model

To build the source model and co-register the individual anatomies to a single template, T1-weighted anatomical MRI scans (TR = 8 ms, TE = 3 ms, 146 slices, 220 mm FOV, 256×256 matrix, 0.9x.0.9×1.0 mm voxels) were acquired for each participant using a GE 3-Tesla Signa MR System (Chicago, United States). Co-registration between MEG and MRI was performed using fiducial-based alignment supplemented with head–shape matching. Each MRI scan was warped to a template defined in a normalized space to allow for group analysis of functional data. Lead fields were computed using a single-shell volume conduction model (Nolte, 2003) derived from each participant’s segmented MRI scan. This model was chosen for its numerical stability and validity, given the magnetic transparency of the skull. Individual MRI scans were spatially normalized to the MNI template using non-linear spatial normalization (Ashburner and Friston, 2005) to facilitate group-level averaging. The source space comprised a regular 3D grid with 5-mm spacing between adjacent nodes.

#### Data processing

The gradiometer data from the MI task and the two control conditions (resting state and mental counting contrasts) were segmented into 1-s trials. Then, imagery and control epochs were inspected for artifacts using different statistics such as variance and kurtosis. Trials were removed if they appeared as gross outliers upon visual inspection of the summary statistics (variance and kurtosis) across channels; epochs containing significant ocular or muscle artifacts identified by this screening were rejected. The mean epoch rejection rates were similarly low across all conditions: imagery 7.0% (median 4.3%, range 0.7–21.7%), counting 6.6% (median 4.0%, range 1.7–16.3%), and resting 5.2% (median 3.1%, range 1.7–15.6%). This data-adaptive approach was chosen over fixed amplitude thresholds to account for inter-individual variability in baseline noise levels.

Power spectra were then computed for each condition, and the average power across all sensors was calculated. The log ratio of power between conditions, in decibels, was used to depict changes in sensor space as topographic maps across frequencies, as described previously (Garcia Dominguez et al., 2023). To determine the frequency of interest (FOI), we calculated the average power changes across all sensors and performed paired *t*-tests at each frequency bin (1–60 Hz). This data-driven approach identified a continuous range of significant differences (*p* < 0.05) between 15 and 21 Hz in the beta frequency band, centered at 18 Hz for both contrast conditions. We expressly used this maximization strategy to optimize the sensitivity of the spatial beamformer to the strongest observed physiological signal, rather than to test a hypothesis regarding beta band specificity. In this study, at the FOI, the MI condition showed a notable desynchronization (power decrease) relative to both the mental counting control and the resting state baseline.

#### Inverse model

A frequency-domain beamformer—dynamic imaging of coherent sources (DICS; Gross et al., 2001)—was then calculated at the FOI over the warped 3D grid. A common spatial filter was computed by pooling imagery and control conditions. This filter was then applied to each condition separately, resulting in a power estimate for each participant, node, and condition (Garcia Dominguez et al., 2023). Since each node is mapped to the template brain, the outcome can be averaged over equivalent nodes across participants and can be plotted on the template brain directly. Because there are two conditions to contrast, we chose to express the beamformer power contrast as the relative change in power of control (resting state or mental counting) versus imagery over every node, calculated as [*P*(control) – *P*(imagery)]/P(imagery), where *P* is power. This formulation allows decreases in power during imagery to appear as positive changes in source space figures. The DICS beamformer and frequency analyses were implemented using FieldTrip (Oostenveld et al., 2011).

#### Statistical analysis

To assess the significance of power changes between conditions, a non-parametric cluster-based permutation test was performed (Maris and Oostenveld, 2007). Given the sample size (*n* = 8), we implemented a custom exact permutation test using MATLAB (R2024b, The MathWorks, Inc.). First, dependent samples t-tests were computed at each voxel of the source grid, comparing the visualization and control conditions. Voxels exceeding a threshold of *p* < 0.05 (uncorrected) were clustered based on spatial adjacency. Spatial clustering was performed on the interpolated 3D source grid (26 × 33 × 28 voxels) using 26-point connectivity (MATLAB bwconncomp function). The cluster-level statistic was defined as the sum of the *t*-values within each cluster. To evaluate significance, a null distribution was generated using all possible permutations of the condition labels (2^8^ = 256 permutations). For each permutation, the condition difference signs were randomly flipped for a subset of participants, and the maximum cluster statistic was recorded. The empirical cluster statistic was considered significant if it exceeded the 95th percentile of this exact null distribution (*p* < 0.05, two-tailed).

## Results

All participants demonstrated good imagery ability on the MIQ-RS (Supplementary material) and reported no difficulty with visualization inside the magnetically shielded room.

The MEG beamforming results obtained at the group level, comparing relative change in power at the FOI for the different conditions (imagery versus resting state or mental counting), revealed prominent desynchronization—that is, decreased oscillatory beta power, reflecting neuronal activation (Miller et al., 2014)—during the imagery condition in the left posterior hemisphere. This effect was maximal around the intraparietal sulcus and extended to the inferior aspect of the superior parietal lobule and the superior aspect of the inferior parietal lobule.

**Figure 1:**
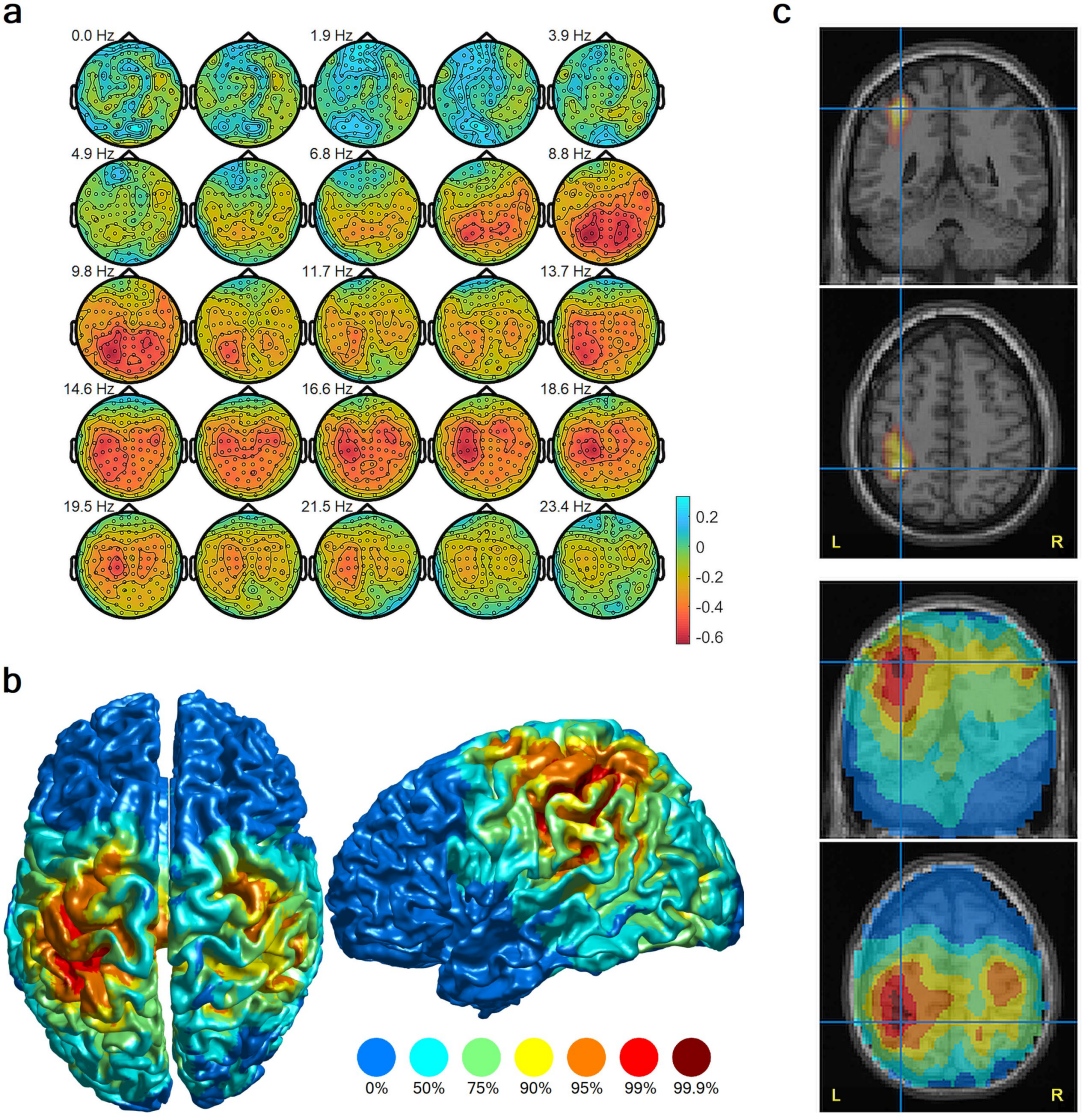
Localization of neural activation during complex ice hockey motor imagery, group-level results, resting state contrast. **(a)** Topographic plots of average log-transformed spectral power differences in sensor space. Red indicates decreased power (neuronal activation) during imagery. **(b)** Cortical surface rendering of MEG beamformer results shows maximal activation in the left intraparietal sulcus and adjacent parietal association cortex and, to a lesser extent, pre- and post-central gyri. Color scales indicate percentiles of relative power change. **(c)** Source solution maximum from **(b)** is displayed on coronal and axial MRI slices, thresholded at the 99th percentile (top) and unthresholded (bottom).

Figure 1 shows the contrast between imagery and the resting state baseline control.

**Figure 2:**
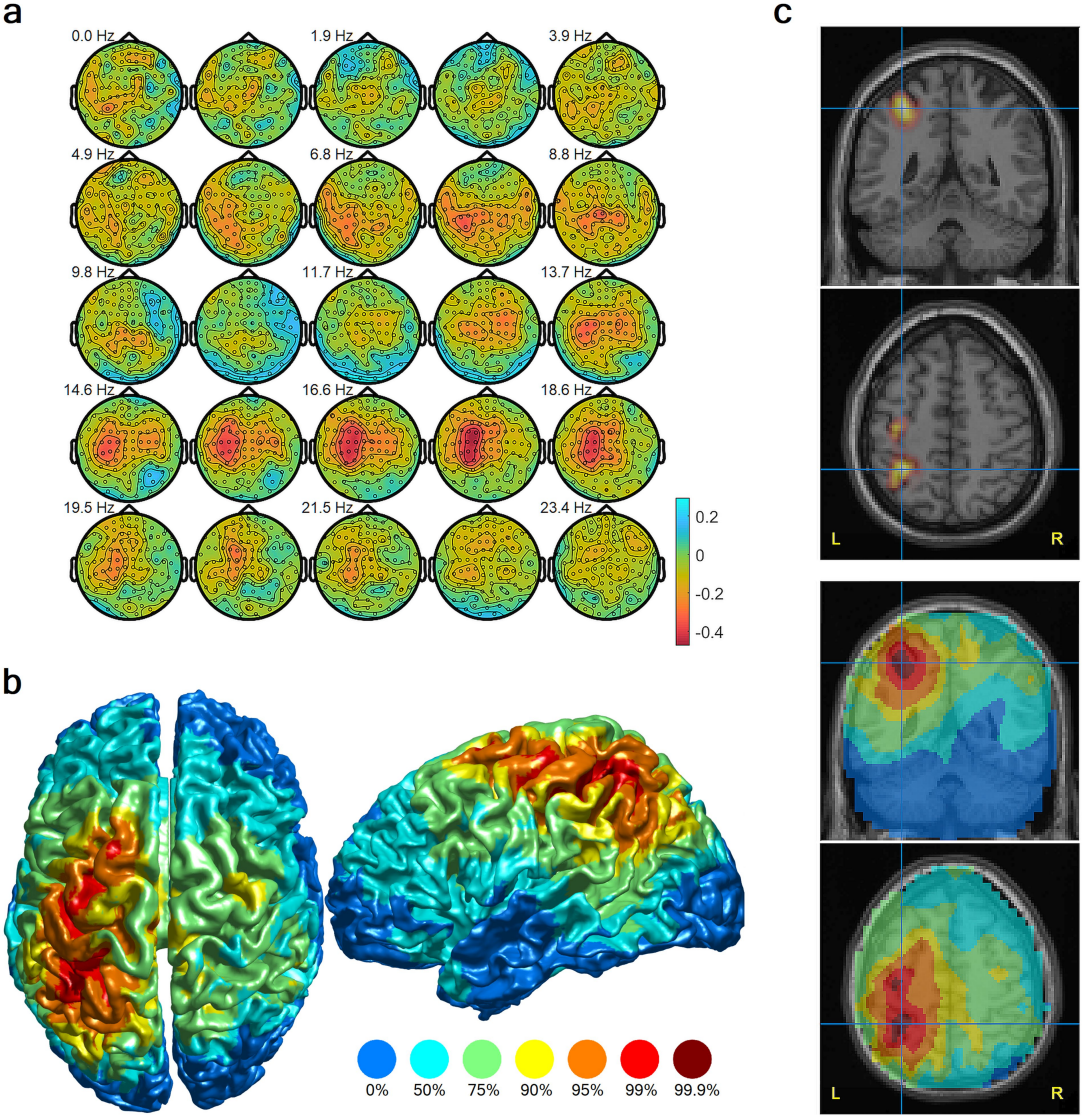
Localization of neural activation during complex ice hockey motor imagery, group-level results, mental counting contrast. **(a)** Topographic plots of average log-transformed spectral power differences in sensor space. Red indicates decreased power (neuronal activation) during imagery. **(b)** Cortical surface rendering of MEG beamformer results shows maximal activation in left parietal association cortex and sensorimotor primary cortex. **(c)** Source solution maximum from **(b)** displayed on coronal and axial MRI slices, thresholded at the 99th percentile (top) and unthresholded (bottom). Color scales indicate percentiles of relative power change.

Figure 2 shows the contrast between imagery and the mental counting control.

The statistical analysis confirmed the observed visualization-related desynchronization to be significant in amplitude and spatial extent in both contrast settings. Specifically, the cluster-based permutation test identified a significant cluster in the posterior left hemisphere parietal region (*p* < 0.01, corrected for multiple comparisons).

As suggested in the group-level figures, along with the maximal left parietal activation, significant desynchronization was also present during the imagery condition in the left > right pre- and post-central gyri. This activation in primary sensorimotor cortex was most evident when the imagery condition was compared to the mental counting control, as opposed to the resting state, implying that the resting state contained greater baseline sensorimotor neural activity than the more focused non-motor mental counting state.

Remarkably, the same pattern of maximal activation in left posterior hemisphere cortex was evident at the individual level in 100% of subjects (8 out of 8 participants). This consistent finding included the goaltender, who used a different visualization script, indicating that the left parietal locus of activation was not dependent on the exact content details of an individual’s MI but rather reflected the dynamic cognitive process of the visualization.

**Figure 3:**
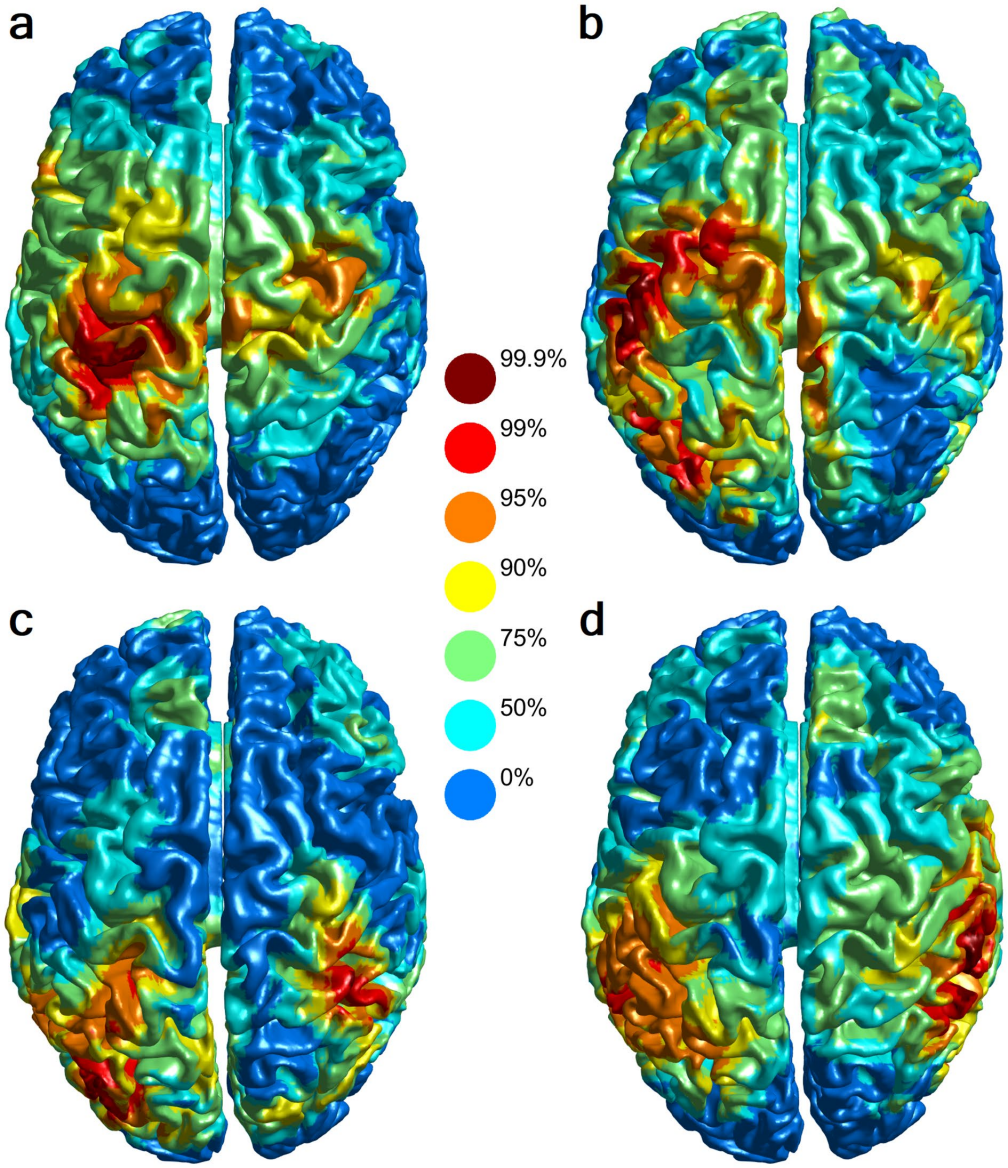
Individual-level MEG source localization during motor imagery versus mental counting control. **(a)** Participant 4, professional, 25 years old. Left parietal activation is maximum with comparatively weaker activation in the sensorimotor cortices. **(b)** Participant 2, NCAA, 25 years old. Left parieto-occipital and sensorimotor cortex activation in the sensorimotor cortices. **(c)** Participant 3, U Sports, 20 years old. Left parieto-occipital activation is greater than right parietal activation. **(d)** Participant 5, NCAA, 21 years old, goaltender (different visualization script). Left parietal activation is greater than right parietal activation. All panels display beamformer source contrast maps color-coded by percentile of relative power decrease.

At the individual level, lesser significant activation in the left and/or right sensorimotor cortex was observed in six out of eight participants (three unilateral left, two bilateral, and one unilateral right). Some of the athletes also showed lesser significant activation in adjacent left occipital cortex (two out of eight participants) and/or in contralateral right parietal cortex (two out of eight participants, most apparent in the goaltender, Figure 3).

## Discussion

Complex PETTLEP-guided visualization in this group of elite female ice hockey players was primarily associated with functional activation of left hemisphere parietal lobe areas involved in somatosensation, kinetic visuospatial processing, and multimodal sensorimotor integration (Farah, 1989; Sirigu et al., 1996). A similar activation pattern was evident when the MI task was contrasted against resting state or active counting conditions, indicating its specificity to the cognitive operations involved in this type of visualization. Despite, or perhaps because of, the complexity of the MI, this region was reliably activated in all participants and could be identified by MEG even at the individual level. Moreover, the same left parietal region was maximally activated in the goaltender, who used a different visualization script, demonstrating that it was not the precise imagery content (beyond being a dynamic multisensorial hockey-specific moving image) but rather the conscious performance of complex MI—the imagining process itself—that was detected by MEG beamforming.

Previous studies of MI using positron emission tomography (PET) or functional MRI (fMRI) have identified multiple additional cortical and subcortical regions of brain activation, including frontal premotor and supplementary motor areas, the cingulate gyrus, the caudate nucleus, and the cerebellum (Hétu et al., 2013; Jiang et al., 2015; Filgueiras et al., 2018), all areas that showed no significant activation identifiable by MEG beamforming. The differences in localization across studies and imaging methods could conceivably reflect differences between complex and simple MI, between open and closed sports, between good imagers and less adept individuals (Guillot et al., 2008), or perhaps especially between PET/fMRI and MEG beamforming.

Our use of MEG beamforming provided a direct measure of neural activity with millisecond temporal resolution, different from PET or fMRI, which could explain the more discrete localization found with MEG, as non-parietal areas identified by PET/fMRI could represent downstream network connections of the principal locus of image generation. The fast temporal resolution of MEG, combined with the short sliding window of the beamforming technique, may have been ideal for the identification of consistent, prolonged activations during visualization. In this context, the beamformer, in essence, acts as a low-pass filter to de-emphasize transient or spatially inconsistent activations that might be more likely to appear as metabolic changes in PET or fMRI (see Supplementary material for more discussion of this issue).

It is conceivable that complex MI in elite hockey players would not significantly activate anterior motor planning areas if the athletes subconsciously “filter out” or automatize the basic motor skills they are already expertly familiar with during visualization. Experienced athletes expend less energy and activate fewer brain areas than novice athletes during MI and sport-related decision-making (Ross et al., 2003; Milton et al., 2007; Filgueiras et al., 2018). At the elite level, where these differences would be expected to be most pronounced, complex MI may be limited largely to the cognitive aspects of imagination and localized primarily to posterior left hemisphere parietal lobe cortex. Dynamic sensorimotor integration in this left parietal region during visualization could facilitate novel connections and optimize the cognitive hub of existing performance-related cortical–subcortical neural networks, providing a possible mechanism whereby covert PETTLEP-guided thought can improve an athlete’s subsequent performance.

The MEG identification of a left parietal cortical hub for complex MI provides both neurophysiological and neuroanatomical support for certain theoretical models derived from cognitive neuroscience; specifically, hierarchical internal forward (Tian and Poeppel, 2010) and predictive-processing (Ridderinkhof and Brass, 2015) models, which propose that “efference copies” or “emulations” of imagined actions are generated and dynamically updated in posterior parietal cortex, upstream from frontal motor planning and subcortical motor coordination areas. Viewed in terms of network connectivity, the continuously active parietal integration hub may transiently recruit other areas not needing sustained activation during imagery, but these areas would either be unidentified by MEG beamforming or show only mild or individual-level activation (e.g., primary sensorimotor, left occipital, or right parietal cortices).

Our findings, while preliminary, are hypothesis-generating, in particular about the brain mechanisms underlying image generation, dreaming, and phenomenal consciousness. Historically, neuropsychological lesion studies using componential analyses to identify focal deficits in image generation found a significant association with lesions in the left posterior hemisphere (Farah, 1984; Stangalino et al., 1995). Interestingly, some of the affected patients also reported a loss of dreaming (Farah, 1984), and a left posterior hemisphere lateralization of dreaming was later supported in a neurological review of these and other cases (Greenberg and Farah, 1986).

Dreaming is quite obviously a natural sleep analog to complex MI, and our demonstration of a similar neuroanatomical localization for complex MI supports and refines the classical imagery localization hypotheses. Localization of the neural correlates of dreaming has been a subject of interest in modern consciousness studies, with the identification of a posterior hemisphere “hot zone” for dreaming used to support the postulate of a similar neural locus for all phenomenal consciousness (Tononi et al., 2016; Siclari et al., 2017). Complex MI is a form of phenomenal consciousness that applies covert thought as a tool to alter subsequent motor behavior. The means by which thought can later influence human behavior, whether in the case of PETTLEP-guided MI for sports performance or as long-reported anecdotally in the case of dreaming, is an unresolved mind–brain problem. Nevertheless, the physical substrate of the process appears to predominantly involve the posterior cortex of the left hemisphere.

### Limitations

Our exploratory study aimed simply to determine if MEG could reliably detect evidence of localized neural activity during PETTLEP-guided complex MI. It was neither conceived as a pilot study nor as a feasibility study aiming to answer other hypothesis-driven questions. For example, it was not intended to compare putative differences in activation patterns between complex and simple MI, open and closed sports, or expert and novice athletes and imagers. The small and unusually homogeneous convenience sample of elite female hockey players experienced in visualization was purposely chosen to maximize the chance of identifying a visualization-related MEG signal; the robustness and reliability of the beamforming results were unexpected. It must be emphasized that the presented findings are limited to this specific type of MI and expert individual, and it cannot be presumed that similarly robust and reliable MEG localization would be found in other athletes or in the general population. Nevertheless, although this was not our aim, our results could be taken as support for the feasibility of using the same MEG beamforming methods in future studies designed to compare activation patterns across different types of MI or in athletes and non-athletes with varying levels of expertise.

## Data Availability

The raw data supporting the conclusions of this article will be made available by the authors, without undue reservation.

## Appendix: Visualization script

### Set up: perspective in first person

Imagine yourself playing a game against an important opponent (choose an opponent). Imagine yourself in the uniform and your equipment. Imagine the feeling of being dressed and holding your stick. Imagine you are in the third period of this game and the score is tied 1–1. Imagine yourself sitting on the bench watching the play go by, waiting for your next opportunity to go out and sacrifice for the team. You are sweaty, breathing heavily, and your adrenaline level is high. Picture the sounds of the crowd, your coach, and your teammates around you. Imagine the cool air from the rink hitting the sweat on your skin.

Imagined scenario (*what participants imagined during the “GO” cues**)

Your coach calls you to play and you hop over the boards as your player changes. You are on the (left or right depending on your handedness) wing when your defender passes you the puck as you begin to skate toward the opponent’s zone. You cradle the puck with ease and pick up speed. You are flying down the ice handling the puck on your blade with your head up, reading the play and the players around you. You see that you are on a 2 vs. 1 with your other teammate against the defender on the opposing team. Your heart is pumping, but you are composed, ready to seize the moment. At this point, you are at the opposing team’s blue line and you have to make a play. You notice their defender cheats toward your player that is on the two-on-one with you. You decide to fake the pass to your teammate, turning your head and stick toward them to fake out the goalie and the defender, but at the last second you turn toward the net and fire a hard shot using your muscle and power high and up to the top (left for right-handed players or right for left-handed players) corner for a goal. The crowd jumps to their feet, you feel a rush of excitement and joy as you throw up your hands to celebrate the goal, skating over to your teammates for a celebratory hug/bear tackle.

Reminders:

- Try to imagine the situation in its entirety, with as much detail and physiological awareness as possible.
- Imagine the sounds, sensations, smells, tastes, emotions, and sights. Channel your focus, heartbeat, sweat, adrenaline, excitement, breath, physical movements, muscle tension, strength, balance, power, the puck on your stick, your hands around the stick, the skates cutting into the ice, your teammates calling, your coaches talking, the crowd cheering, the wind in your face and hair, the deception in your body positioning and any other details that relate to the visualized situation.
- Keep the PETTLEP model in mind: physical, environment, task, timing, learning, emotion, perspective.

*One participant, a goaltender, imagined a different scenario specific to her position. The imagery was a penalty-killing situation (her team playing short one player), with the puck controlled by the opposition team in her own team’s zone, the puck moving from one opponent to another through a series of passes in a structured power play formation, her own re-positioning movements mirroring the changing location of the puck handler. The imagined play culminates in a final cross-ice pass just in front of the goal crease and a one timer shot that she saves after a strong lateral push and power slide, having read the play perfectly, allowing no rebound.

The goaltender’s scenario presumably differed most from the other participants’ scenario in the task and learning elements of the PETTLEP model (and possibly the emotion element, if preventing a goal is more emotionally taxing than scoring a goal, which is likely in hockey).
