# Supplementary material for: Complex motor imagery in elite female ice hockey players: a cortical arena of imagination revealed by magnetoencephalography
Source: Front Hum Neurosci. 2026 Feb 27;20:1754371. doi: 10.3389/fnhum.2026.1754371 (PMC12982352; doi:10.3389/fnhum.2026.1754371)
Supplement: Supplementary file 1 [file Data_Sheet_1.PDF]

Supplementary Material

1. Movement Imagery Questionnaire—Revised second version (MIQ-RS)

The **MIQ-RS** (Gregg et al., 2010) is composed of two subscales, visual and kinesthetic, each represented by seven items. Participants rate the ease or difficulty of imagining the movement on a 7-point scale from 1 = very hard to see/feel to 7 = very easy to see/feel.

Motor imagery: involves vivid mental rehearsal of complex or simple movements without motor execution

- **Kinesthetic motor imagery** → feeling what performing a movement is like without actually doing the movement
- **Visual motor imagery** → forming a visual image or picture of a movement in your mind

Participant MIQ-RS scores

Participant	Level	MIQ-RS Visual Imagery Score	MIQ-RS Kinesthetic Imagery Score	MIQ-RS Total Score
1	NCAA	42/49	42/49	84/98
2	NCAA	32/49	40/49	72/98
3	U Sports	33/49	32/49	65/98
4	Olympic / Professional	39/49	41/49	80/98
5	NCAA	43/49	36/49	79/98
6	Professional	39/49	38/49	77/98
7	U Sports	40/49	43/49	83/98
8	U Sports	45/49	44/49	89/98

Reference

Gregg, M., Hall, C., and Butler, A. (2010) The MIQ-RS: A suitable option for examining movement imagery ability. *Evid. Based Complement. Alternat. Med.* 7: 249-257. https://doi:10.1093/ecam/nem170

2. MEG beamforming versus PET or fMRI localization of activation

MEG is relatively insensitive to neural activity in subcortical or cerebellar sources compared to PET and fMRI. MEG beamforming also works on a different timescale than PET or fMRI, and fast changes in neuronal activity directly detected using MEG may be relatively diluted in the indirect metabolic measurements of PET or fMRI.

The DICS inverse model we employed for MEG localization used a cross-spectral density matrix in the beta band (13-30 Hz), sliding 1-second windows, with 50% overlap, across the entire 30-second block. In practice, this means that every voxel was weighted by how deeply and for how long its beta power was suppressed during each 30-second imagery block. This method inherently favors consistent, long-lasting neuronal activation/beta desynchronization: activation sustained across many consecutive 1-second segments will be identified by the beamformer as a strong source of power contrast. Conversely, regions activated only briefly or sporadically during complex MI will be “washed out” in the averaging of spectral power. Averaging across 10 trials per participant (and, for the group-level analysis, averaging across participants) further amplifies this effect. In essence, the 1-second sliding window of the beamforming technique acted as a low-pass filter that emphasized prolonged activations and de-emphasized transient activations, the latter more likely to be identified as metabolic changes by PET or fMRI. The main parietal hub region of neural activation identified by MEG was desynchronized for a large fraction of each imagery block, and so its signal added up, window after window, to become the strongest peak in the final activation map.

Other, downstream areas may conceivably have had beta power decreases during imagery periods, but for briefer periods of time or in slightly different voxels from one participant to the next. The contributions of these other areas of desynchronization to the overall pattern of neural activation identified by MEG may have been intermittent and/or spatially inconsistent, thus adding only a small amount of power to the group-level average. Spatial inconsistency could occur given that participants are imagining a complex and kinesthetic scene – sweat, noise, different types of movements – which may be processed in different areas in different participants and end up diluted in the grand average. However, as the parietal hub integrates all these modalities, it would remain desynchronized throughout the imagery periods.
